# Pharmacological inhibition of Carbonic Anhydrase IX and XII to enhance targeting of acute myeloid leukaemia cells under hypoxic conditions

**DOI:** 10.1111/jcmm.17027

**Published:** 2021-11-17

**Authors:** Fangli Chen, Emilia Licarete, Xue Wu, Daniela Petrusca, Callista Maguire, Max Jacobsen, Austyn Colter, George E. Sandusky, Magdalena Czader, Maegan L. Capitano, James P. Ropa, H. Scott Boswell, Fabrizio Carta, Claudiu T. Supuran, Brian Parkin, Melissa L. Fishel, Heiko Konig

**Affiliations:** ^1^ Melvin and Bren Simon Comprehensive Cancer Center Indiana University Indianapolis Indiana USA; ^2^ Department of Molecular Biology and Biotechnology Faculty of Biology and Geology Babes‐Bolyai University Cluj‐Napoca Romania; ^3^ Department of Pathology and Laboratory Medicine Indiana University Indianapolis Indiana USA; ^4^ NEUROFARBA Department Pharmaceutical and Nutraceutical Section University of Florence Firenze Italy; ^5^ Division of Hematology and Oncology Department of Internal Medicine University of Michigan Ann Arbor Michigan USA; ^6^ Department of Pediatrics Wells Center for Pediatric Research Indiana University Indianapolis Indiana USA; ^7^ Department of Pharmacology & Toxicology Indiana University Indianapolis Indiana USA

**Keywords:** acute myeloid leukemia, Carbonic Anhydrases, drug resistance, hypoxia, pH regulation

## Abstract

Acute myeloid leukaemia (AML) is an aggressive form of blood cancer that carries a dismal prognosis. Several studies suggest that the poor outcome is due to a small fraction of leukaemic cells that elude treatment and survive in specialised, oxygen (O_2_)‐deprived niches of the bone marrow. Although several AML drug targets such as FLT3, IDH1/2 and CD33 have been established in recent years, survival rates remain unsatisfactory, which indicates that other, yet unrecognized, mechanisms influence the ability of AML cells to escape cell death and to proliferate in hypoxic environments. Our data illustrates that Carbonic Anhydrases IX and XII (CA IX/XII) are critical for leukaemic cell survival in the O_2_‐deprived milieu. CA IX and XII function as transmembrane proteins that mediate intracellular pH under low O_2_ conditions. Because maintaining a neutral pH represents a key survival mechanism for tumour cells in O_2_‐deprived settings, we sought to elucidate the role of dual CA IX/XII inhibition as a novel strategy to eliminate AML cells under hypoxic conditions. Our findings demonstrate that the dual CA IX/XII inhibitor FC531 may prove to be of value as an adjunct to chemotherapy for the treatment of AML.

## 1 INTRODUCTION

Acute myeloid leukaemia (AML) represents a heterogeneous, malignant disorder of the haematopoietic system that is characterised by rapid cell growth, dysregulated apoptosis and impaired differentiation of leukaemic blasts.^1^ While standard anti‐neoplastic therapies, including recently approved targeted agents (e.g. inhibitors of FLT3 and IDH1/2), and allogeneic stem cell transplantation frequently lead to remissions, most patients relapse due to the development of drug resistance and minimal residual disease (MRD).^1^, ^2^ The substrate of MRD is the survival of a small drug‐resistant population of leukaemic cells in specific “sanctuaries” such as the bone marrow (BM) niche.^3^, ^4^, ^5^ Recent data suggests that distinct regions of the BM niche are severely deprived of oxygen (O_2_).^6^, ^7^, ^8^, ^9^ Although factors such as BM stromal cells and Hypoxia Inducible Factor (HIF) signalling have been implicated in niche‐associated leukaemic cell survival and MRD development, therapeutic approaches to disrupt leukaemic cell‐BM stromal interaction and HIF‐targeted strategies have yielded limited success to date.^10^, ^11^, ^12^, ^13^, ^14^, ^15^, ^16^, ^17^, ^18^ As a result, there remains a significant knowledge gap regarding the most relevant targets in AML.

Carbonic Anhydrases (CA) IX and XII have recently emerged as attractive targets in multiple tumours.^19^, ^20^, ^21^, ^22^ CAs function as transmembrane proteins that mediate intracellular pH under low O_2_ conditions via the reversible conversion of CO_2_ to bicarbonate and protons.^23^ Lines of evidence suggest that maintaining a close to neutral, intracellular pH represents a key survival mechanism for tumour cells to escape apoptosis and to proliferate in the hypoxic environment.^24^ Confined to few normal tissues, CA IX is highly expressed in many solid tumour types and has been associated with poor prognosis, disease progression and aggressiveness.^25^, ^26^ A therapeutic effect of CA IX inhibition has been demonstrated in preclinical in vivo studies of renal cell and colorectal cancer where specific inhibition of CA IX activity enhanced the effects of tumour irradiation.^27^, ^28^ However, the role of CA IX in AML is not well defined. Recent work by Konopleva et al. demonstrated that CA IX expression strongly correlates with the BM blast percentage in AML patients, indicating that CA IX stabilisation is closely associated with the extent of leukaemic infiltration.^29^ Compared to CA IX, data on the role of CA XII in human cancers is less abundant. Similar to CA IX, CA XII expression has been shown to be upregulated in several solid malignancies.^30^, ^31^, ^32^, ^33^ In addition, Lounnas et al. as well as others demonstrated that CA XII promotes cancer cell survival, migration, invasion and stemness.^34^, ^35^, ^36^ Further work by Kopecka and colleagues strongly suggests that targeting CA XII has the potential to overcome drug resistance as increased expression of CA XII has been found on the surface of chemo‐resistant cells.^19^

We herein evaluated the expression of CA IX and XII in AML cells under O_2_‐controlled settings and uncovered a potential role for CA IX and CA XII in AML cells exposed to hypoxic and chemotherapeutic stress conditions. We therefore visited the approach of dual CA IX and XII inhibition in a diverse panel of AML cells and in patient derived (PDX) AML models. Our data demonstrates that dual CA IX/XII inhibition exerts anti‐leukaemic activity *in vitro* and *in vivo*, is well tolerated in single agent mode and when combined with Cytarabine, and improves survival. On the basis of our findings, we propose the further investigation of integrated CA IX/XII targeting in AML.

## 2 METHODS

### 2.1 Cell culture and reagents

Molm13 (M13), Molm14 (M14) and the relatively Cytarabine‐resistant cell lines OCI‐AML3 and THP‐1^37^ were obtained from the Deutsche Sammlung von Mikroorganismen und Zellkulturen (Braunschweig, Germany). All cells were cultured in RPMI1640 medium with 10% FBS and 100 U/mL streptomycin/penicillin in a humidified atmosphere at 37°C and 5% CO_2_. Quizartinib and Cytarabine were purchased from Selleck Chemicals, dissolved in dimethyl sulphoxide (DMSO) at stock concentrations of 10 mM and stored at −80°C. The dual CA IX/XII inhibitors FC531 and SLC0111 were synthesised as previously described.^22^ The dual CA IX/XII inhibitor CA912 was purchased from Calbiochem (Calbiochem Research Biochemicals, Burlington, MA). All CA IX/XII inhibitors were dissolved in DMSO at stock concentrations 10 mM and stored at −80°C. Fluorescein isothiocyanate‐AnnexinV antibody and propidium iodide were purchased from BD Pharmingen (556420) and Sigma‐Aldrich (P4864), respectively. APC‐CD11b antibody was purchased from Biolegend (101212).

### 2.2 Patient samples

AML blasts derived from whole blood or the BM from newly diagnosed (ND) and relapsed/refractory (R/R) AML patients (pts) were obtained under guidelines approved by the Institutional Review Board of the Indiana University (Indianapolis, IN, USA). All patients gave informed consent according to the Declaration of Helsinki. Blasts were separated as described elsewhere.^38^ The clinical characteristics of the patients from whom samples and tissues were derived are listed in Tables 1A and 1B.

**TABLE 1A:** Patient characteristics (FLT3 mutated AML patients)

Patient No.	Age (years)	Gender, male (M)/female (F)	Newly diagnosed (ND), Relapsed/Refractory (R/R)	Cytogenetics	Molecular alterations identified
1	33	F	ND	46,XX,inv(12) (p11.2q13)	FLT3/ITD, NPM1
2	75	F	ND	46,XX	FLT3/ITD, NPM1
3	24	F	ND	47, XX, +8	FLT3/ITD
4	56	M	ND	46, XY	FLT3/ITD, ATRX, CCT6B, KMT2C (MLL3), NPM1, PTPN11, RAD21
5	50	F	ND	46, XX	FLT3/TKD, IDH2, DNMT3A
6	35	F	R/R	46, XX	FLT3/ITD, NPM1
7	60	M	R/R	46, XY	FLT3/ITD
8	36	F	R/R	46, XX	FLT3/ITD, NPM1, ATRX, ASXL1, RUNX1, TET2
9	30	M	ND	46, XY	FLT3/ITD, WT1
10	39	M	ND	46,XY,t(5;22)(q35;q11.2)	FLT3/ITD, NRAS, PDCD11, WT1
11	46	M	NDx	47,XY,+11	FLT3/ITD, TET2, KMT2A, SRSF2
12	38	F	NDx	46, XX	FLT3/ITD, FLT3/TKD

**TABLE 1B:** Patient characteristics (Non‐FLT3 mutated AML patients)

Patient No.	Age (years)	Gender, male (M)/female (F)	Newly diagnosed (ND), Relapsed/Refractory (R/R)	Cytogenetics	Molecular alterations identified
13	63	M	ND	46,XY,del(20)(q11.2q13.3)[9]/46,sl,del(5)(q13q33),+7,der(7;17)(q10;q10),+8,add(11)(q23),18,3~6dmin[5]/47,sdl1,+6,3~20dmin[2]/46,sdl2,der(7;17),−8,5~12dmin[2]/46,XY[2]	P53, KMT2A, NF1
14	25	M	ND	46, XY	Unknown^*^
15	65	M	ND	45,XY,add(1)(p22),−2,−5,add(9)(p11), −12,−15, add(16)(q22), add(17)(p11.2), −18,+mar1, +mar4,+mar5,+mar8[7]/ 43~44,idem, ‐X,−3,−4,−7, −20,+mar2,+mar3, +mar6,+mar7[cp5]/ 46,XY[8]	P53
16	66	M	ND	45,XX,add(5)(q1?3),add(14)(p12),add(17)(p11.2),−18[2]/45,sl,der(16)t(11;16)(q13;p13.3)[11]/ 46,sdl1,+8[5]/ 46,XX[3]	NRAS, PTPN11, ASXL1, TP53, WT1
17	60	F	ND	92<4n>,XXXX[2]/ 46,XX[18]	IDH2, RUNX1, SF3B1

^*^ Patient declined molecular testing.

### 2.3 MTT cell viability assay

1 × 10^4^ Molm13, Molm14, THP‐1 and OCI‐AML3 suspended in RPMI1640 culture medium with 10% FBS were seeded in 96 well plates. After 24 h pre‐incubation, cells were treated with drug under ambient air (normoxia, 21% O_2_) or hypoxic conditions (1% O_2_) for 48 has outlined for each experiment. For primary cells, 15 × 10^4^ fresh primary cells were suspended in RPMI1640 culture medium with 20% FBS. Cell viability was assessed using MTT (3‐(4,5‐dimethylthiazol‐2‐yl)‐2,5‐diphenyltetrazolium bromide; Roche, Indianapolis, IN, USA) in accordance to the manufacturer's recommended protocols. Unless otherwise specified, all assays were performed in *triplicate* and results were obtained in at least three independent experiments.

### 2.4 Apoptosis assay

5 × 10^5^ leukaemia cells were seeded in six well plates for 24 h pre‐incubation, and incubated with Cytarabine, Quizartinib or FC531 under 21% or 1% O_2_ conditions for 48 h. Leukaemia cells were collected from the plates, washed twice with cold PBS and then re‐suspended in 100 μl binding buffer. 5 μl FITC‐Annexin V and 10 μl propidium iodide stock solution (50 µg/ml) were added to each sample and incubated at room temperature in the dark for 15 min. Cells were analysed on a FACSCalibur machine (BD Biosciences, San Jose, CA) and the data was analysed by FlowJo software.

### 2.5 Differentiation assay

5 × 10^5^ leukaemia cells were pre‐incubated in six well plates for 24 h and treated with different concentrations of FC531 under 1% O_2_ conditions for 72 h. Leukaemia cells were collected from the plates, washed twice with cold PBS and then re‐suspended in 100 μl PBS. 5 μl APC‐CD11b antibody were added to each sample and incubated at room temperature in the dark for 30 min. CD11b expression was analysed on a FACSCalibur machine (BD Biosciences, San Jose, CA) and the data was analysed by FlowJo software.

### 2.6 Intracellular pH assay

Intracellular pH was evaluated using the pHrodo Red AM Intracellular pH Indicator (Thermo Fisher Scientific, Waltham, MA). Molm14 cells treated with FC531 under normoxic and hypoxic conditions for 48 h were analysed with pHrodo Red AM dye. Results were normalised to cell viability by MTT assay. Intracellular pH calibration buffers were used to create a standard curve of fluorescence intensity for determination of pH values.

### 2.7 RNA isolation and QPCR

Molm13, Molm14, THP‐1, OCI‐AML3 and primary cells were incubated under normoxic or hypoxic conditions for 48 h. Total RNA was extracted by RNeasy Plus Kit (Qiagen) according to the manufacturer's instructions. cDNA was synthesised with reverse transcription kit (Thermo Fisher Scientific) in accordance with the manufacturer's protocols. The PCR primers used in the study were as follows:

- CA IX(F): GTGCCTATGAGCAGTTGCTGTC and CA IX(R): AAGTAGCGGCTGAAGTCAGAGG
- CA XII(F): GACCTTTATCCTGACGCCAGCA and CA XII (R): CATAGGACGGATTGAAGGAGCC
- CA I(F): TAGTGTCTCCTACAACCCAGCC and CA I(R): GCTGTCAGAGAAAGGACCACCT
- CA II(F): GTGACCTGGATTGTGCTCAAGG and CA II(R): GTTGTCCACCATCAGTTCTTCGG
- RN18S1(F): ACCCGTTGAACCCCATTCGTGA and RN18S1 (R): GCCTCACTAAACCATCCAATCGG

RN18S1 expression was used as a control for RNA loading. Quantitative PCR reactions were performed using PowerUp SYBR Green PCR Master Mix (Applied Biosystems, USA) on 7900HT Real‐Time PCR System.

### 2.8 Droplet Digital (dd) PCR

Genomic DNA from circulating leucocytes was extracted from murine whole blood using QIAamp DNA Micro columns (Qiagen) and amplified with a REPLI‐g Single Cell kit (Qiagen). An allele‐specific fluorescent locked nucleic acid probe set (IDT, Coralville, IA) for the FLT3‐ITD of interest was developed and optimised. ddPCR was then performed using that probe set with the murine leucocyte genomic DNA samples along with wildtype (negative) and mutant (positive) controls on a BioRad RainDrop system as previously described.^39^

### 2.9 Animals and establishment of tumour xenografts

All animal studies were performed according to an IACUC‐approved protocol and in compliance with the Guide for the Care and Use of Laboratory Animals (National Research Council, 2011). In brief, Molm14 and PDX xenografts were generated by injecting 2 × 10E5 and 1 × 10E6 cells into the tail vein of 6–8 weeks old NOD‐SCID^γnull^ (NSG) or NSG‐SGM3 (NSGS, JAX stock# 013062) mice (J000106134), respectively. Upon confirmation of engraftment, mice were randomised into experimental groups (*n* = 5 each group) and dosed accordingly with Cytarabine and/or FC531 starting on Study Day 0. Tumour burden was monitored once weekly by flow cytometry on whole blood samples collected *via* retro‐orbital bleed. The flow cytometry panel included mouse CD45, human CD45, human CD3, human CD33, human CD11b, viability dye and absolute counting beads. All animals were monitored for up to 12 weeks following the last dose of therapy prior to be killed by CO_2_ asphyxiation when moribund.

### 2.10 Immunohistochemistry (IHC), digital imaging and analysis

All mouse tissue samples were collected following a detailed Laboratory Animal Resource Centre approved protocol. Human BM samples were obtained from nine AML patients. Slides were stained with H&E, CA IX and CA XII for pathologic evaluation. All tissue sections were scanned at 20× magnification, unless indicated otherwise. Antigen retrieval was performed by immersing the slides in Target Retrieval Solution (DAKO; Agilent Technologies, Santa Clara, CA) for 20 min at 90°C. Subsequent staining steps were performed on the DAKO Immunostainer. Primary antibodies included anti‐mouse CA IX (Novus NB100‐47) and anti‐mouse CA XII (Abcam ab244309). The Aperio Scan Scope CS system (Leica Biosystems, Wetzlar, Germany) was used for imaging. The Positive Pixel Count algorithm on Aperio ImageScope (Leica Biosystems) was used to quantify the amount of a specific stain present in a scanned slide image.

### 2.11 Statistical analysis

Statistical significance of differences was determined by Student's *t* test. Difference was considered statistically significant when *p*‐value was less than 0.05. Data is presented as mean ± standard error of the mean (SEM) unless stated otherwise. Kaplan–Meier survival curve estimators were reported for each condition. The Cox proportional hazard model was used to test the difference of the time‐to‐event among different conditions.

## 3 RESULTS

### 3.1 Hypoxic stress leads to induction of CA IX and/or CA XII in AML cells

**FIGURE 1:**
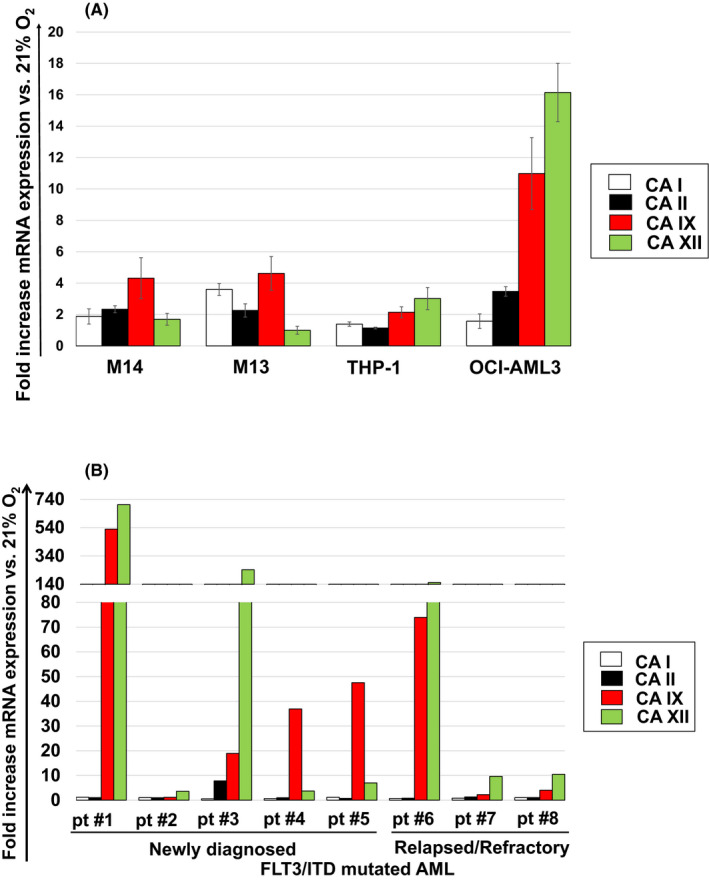
Hypoxic stress leads to the induction of CA IX and/or XII mRNA expression in AML cells. Focussed quantitative RT‐PCR assays for CA I, II, IX and XII were carried out in (A) a diverse panel of AML cell lines as well as in (B) primary cells derived from FLT3/ITD^+^ AML patients with ND and R/R disease after exposure to 21% and 1% O_2_ for 48 h. Results shown in (A) represent the mean ± SEM fold increase in mRNA expression versus the untreated control at 21% O_2_ (*n* = 3). Due to the limited availability of human tissue, primary cell culture experiments (B) were only performed once for each patient sample

Previous work on cancer cells derived from patients with a broad spectrum of solid tumours has shown that hypoxia leads to the induction of complex signalling networks such as the hypoxia‐inducible factor 1α (HIF‐1α) and its downstream targets and transcriptional regulators, including CAs. While these findings have established hypoxia as a compelling target in the treatment of solid cancers, data about hypoxic responses in haematologic neoplasms are lacking. Almost two decades ago, Leppilampi et al. reported that CA II is expressed on leukaemic blasts in 62% of patients with AML.^40^ Aside from the work by these authors, there is paucity of information about the aetiological connection between CAs and AML. To investigate whether CAs are expressed in AML cells under hypoxic stress conditions we exposed a diverse panel of AML cell lines to 21% and 1% O_2_ for 48 h and thereafter assessed the expression of several CAs. Since out of 16 CA isoforms only CA I and II (cytosolic) have previously been reported to be expressed in the haematopoietic system, whereas CA IX and XII (membrane bound) have been linked to solid neoplasms,^41^ we focussed our analysis on these four CAs. Using real‐time quantitative PCR analysis, we found that CA IX and/or CA XII, less so CA I and/or CA II, were highly expressed under hypoxic stress in THP‐1 and OCI‐AML3 as well as in the FLT3/ITD mutated (FLT3/ITD^+^) cell lines Molm13 and Molm14 (Figure 1A). Next, we extended our experiments to include primary samples derived from FLT3/ITD^+^ AML patients with ND (*n* = 5) and R/R (*n* = 3) disease. Our PCR data revealed that, similar to our findings in cell line studies, CA IX and XII, but not CA I and II, were strongly induced under 1% compared to 21% O_2_ conditions (Figure 1B). Of note, FLT3/ITD^+^ AML represents one of the most aggressive forms of blood cancer and carries a dismal prognosis due to short remission durations and high relapse rates.^42^ The dynamics associated with FLT3/ITD^+^ AML indicate that distinct mechanisms of resistance and MRD are operative in this disease, thus making it an ideal model to study the effects of novel therapeutic approaches. Although established as an effective drug target in AML, previous data has shown that FLT3 is downregulated in response to hypoxic stress,^43^ indicating that other signalling pathways influence the ability of AML cells to escape cell death and to proliferate in hypoxic environments.

### 3.2 CA IX and/or CA XII is expressed in Cytarabine‐residual FLT3/ITD^+^ AML cells in vivo

**FIGURE 2:**
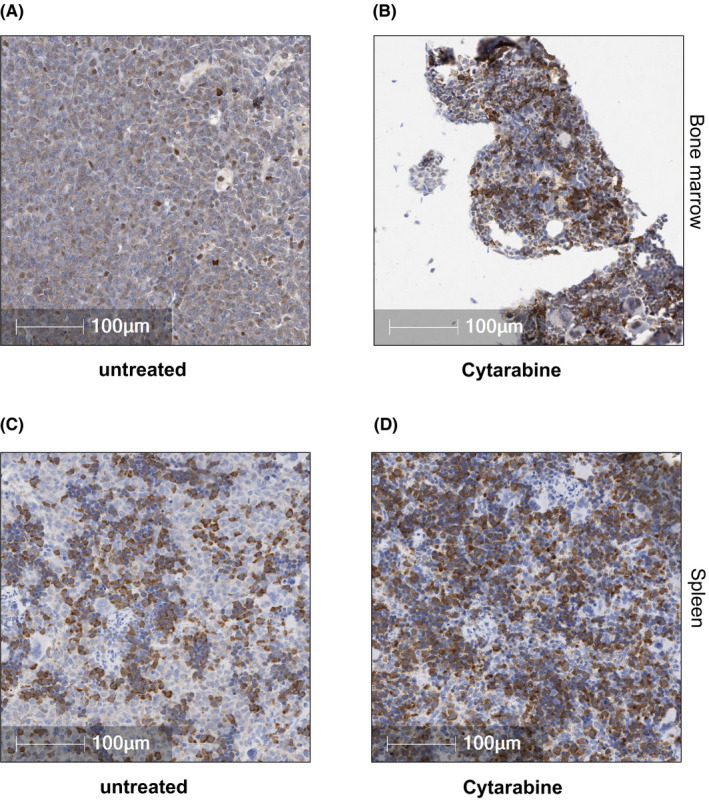
CA IX and/or CA XII is expressed in Cytarabine‐residual FLT3/ITD^+^ AML cells in vivo. (A) Representative tissue sections from untreated animals showed BM hypercellularity with collapse of the vascular channels, occasional small foci of normal erythroid and myeloid cells as well as few megakaryocytes. The vast majority of cells seen in the marrow consisted of large myeloid blasts filling most of the marrow space. The immunostaining for CA IX was multi‐focal and localised on the cell membrane. (B) Treatment of M14 xenografts with Cytarabine (12.5 mg/kg BW) for 5 days led to a reduction in the leukemic burden with enhanced CA IX staining in blasts remaining after treatment with Cytarabine. (C) In untreated animals, the spleen showed marked expansion of the red pulp with large, myeloid blasts replacing most of the normal architecture. (D) In Cytarabine treatment animals, less leukemic blasts were observed in the red pulp compared to the untreated group. Similar to the findings in the BM, CA IX staining was multi‐focal and enhanced in blasts remaining after treatment with Cytarabine. The Positive Pixel Count algorithm on Aperio ImageScope (Leica Biosystems) was used to quantify the amount of CA IX and CA XII staining present in a scanned slide image. A range of colour (range of hues and saturation) and three intensity ranges (weak, positive and strong) were masked and evaluated. The analysis specific to this project required the exclusion of macrophages in the field of analysis. Macrophages stain intensely with CA‐IX and CA‐XII and were therefore excluded from the analysis. An FDA‐approved algorithm was used to distinguish between brown and blue pixels. Corresponding H&E stained sections from the BM and spleen are shown in Figure S2A–D

**FIGURE 3:**
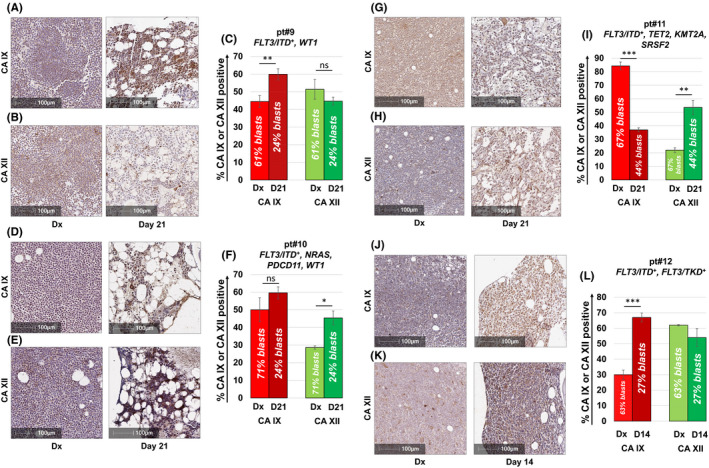
CA IX and/or CA XII is expressed in FLT3/ITD^+^ AML patients failing induction chemotherapy. CA IX and XII staining of BM samples from patients with FLT3 mutated AML (*n* = 4) who had residual disease on day 14 (pt#12, J–L) or day 21 (pt#9–11, A–I) marrow assessment. Consistent with data obtained from M14 xenograft studies, four out of four patients showed increased CA IX and/or CA XII staining in leukemic blasts remaining after induction chemotherapy. Pts #9–11 were treated with an induction regimen consisting of “7+3” combined with the FLT3 inhibitor midostaurin. Pt #12 received induction chemotherapy with “7+3” only as treatment occurred prior to the approval of midostaurin. Results shown represent the mean ± SEM % CA IX or XII positivity. Statistically significant changes in the percentage of CA staining are indicated (* <0.05; **p < 0.01; ***p < 0.001). Leukemic blast cell percentages were quantified per clinical flow cytometric immunophenotyping by the Indiana University Health Pathology Laboratory. Corresponding H&E stained sections from the BM are shown in Figure S3A–H

Following our experiments aimed at assessing CA I, II, IX and XII mRNA expression in AML cells to low O_2_ stress in vitro, we decided to corroborate our findings in xenograft studies. Using Molm14 xenografts, we administered a 7‐day course of Cytarabine and sacrificed mice 24 h after the last dose in order to assess immediate drug responses in vivo. As expected, IHC staining in untreated animals revealed a hypercellular marrow with collapse of the vascular channels. The vast majority of cells seen in the marrow were large myeloid, leukaemic blasts consistent with the development of frank AML. The immunostaining for CA IX was multi‐focal and localised on the cell membrane (Figure 2A). Consistent with our in vitro findings, treatment with Cytarabine reduced the leukaemic burden but CA IX staining was enhanced in residual blasts (Figure 2B). Similar findings were obtained in the spleen. In untreated animals, the spleen was enlarged with marked expansion of the red pulp and large, myeloid leukaemic blasts replacing most of the normal architecture (Figure 2C). The spleen was smaller with less leukaemic blasts in the red pulp compared to the untreated group in animals treated with Cytarabine. Residual blasts in the spleen showed enhanced CA‐IX staining (Figure 2D). Of note, IHC did not reveal relevant CA XII staining, which was consistent with low CA XII mRNA expression of Molm14 cells in PCR assays (Figure 1A). If CA IX and CA XII are involved in the development of AML drug resistance, then patients failing induction chemotherapy as demonstrated by an interim BM assessment routinely performed 14–21 days after the initiation of therapy should show persistent or enhanced CA IX and/or CA XII staining when compared to the diagnostic marrow. We therefore stained BM samples from patients with FLT3 mutated AML (*n* = 4) who had residual disease on their day 14 or day 21 marrow assessment. As shown in Figure 3A–L, four out of four patients showed increased CA IX and/or CA XII staining in leukaemic blasts remaining after induction chemotherapy when compared to the diagnostic marrow.

### 3.3 Dual CA IX/ XII inhibition selectively kills FLT3/ITD^+^ AML cells under hypoxic conditions in vitro

**FIGURE 4:**
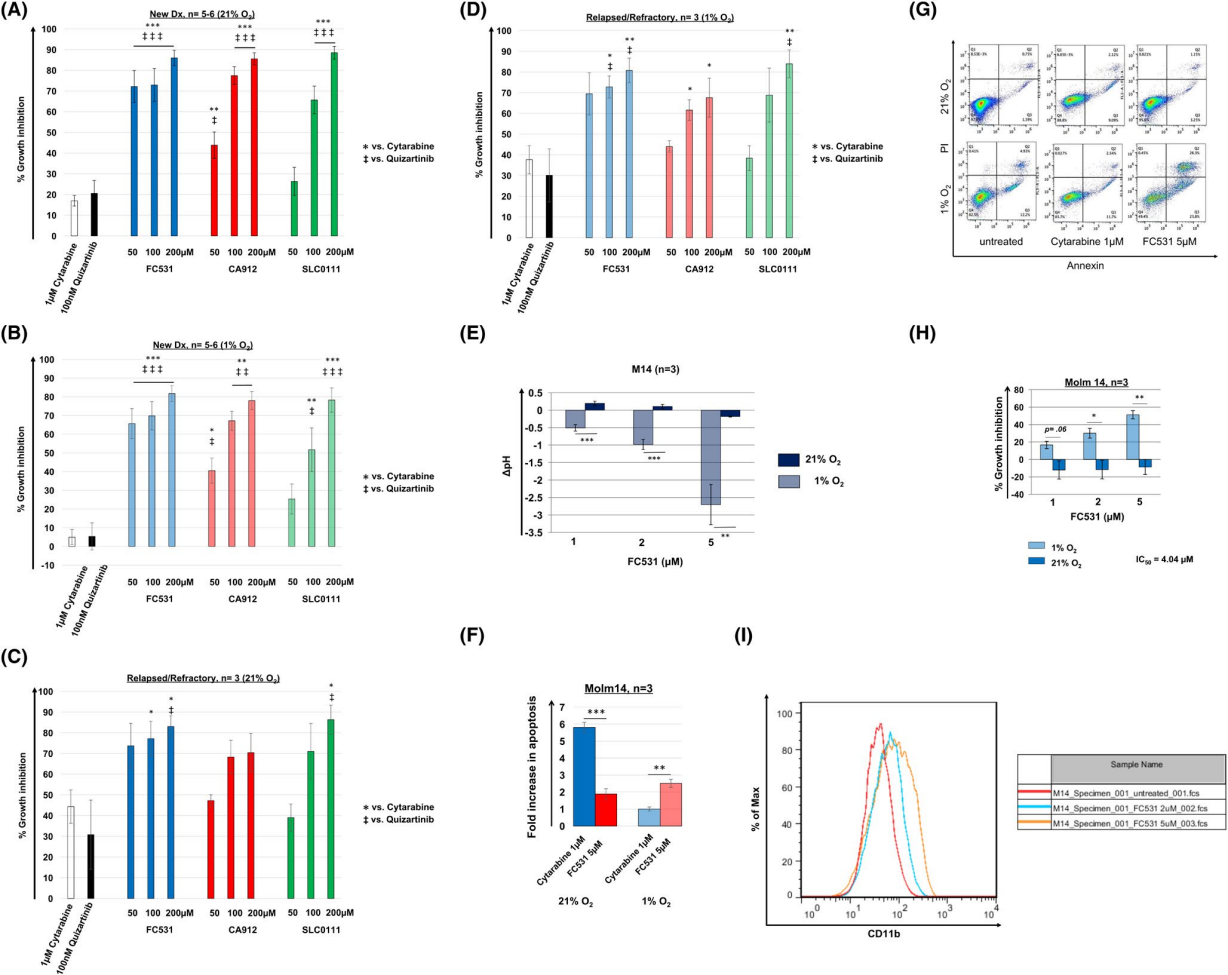
Dual inhibition of CA IX and XII in FLT3/ITD^+^ AML cells leads to intracellular acidification, induction of apoptosis and differentiation under hypoxic conditions. The dual CA IX/XII inhibitors FC531, CA912 and SLC0111 confer growth inhibitory effects under ambient air and hypoxic cell culture conditions in ND (A, B) and R/R (C, D) primary AML patient samples. FC531‐, CA912‐ and SLC‐0111‐induced growth inhibition occurred in a dose dependent fashion and was significantly greater than in response to clinically achievable concentrations of Cytarabine and Quizartinib, which only had mild to no effects in the high and low O_2_ setting. Cells were incubated in the presence of Cytarabine, Quizartinib or dual CA IX/XII inhibitors under 21% or 1% O_2_ for 48 h. Growth inhibition was assessed using MTT cell viability assays. The mean ± SEM is based on replicate experiments (*n* = 5–6 [ND]), (*n* = 3 [R/R]). Statistically significant changes in the percentage of growth inhibition are indicated (* or ‡*p* < 0.05; ** or ‡‡*p* < 0.01; *** or ‡‡‡*p* < 0.001). (E) Dual CA IX/XII targeting with FC531 acidifies the intracellular pH in M14 cells in a dose dependent manner under hypoxic but not under ambient air conditions. The mean ± SEM is based on replicate experiments (*n* = 3). Statistically significant changes in intracellular acidification are indicated (**p < 0.01; ***p < 0.001). (F) FC531 induces apoptosis in M14 cells at 1% O_2_ and is significantly more effective than Cytarabine in this respect. Apoptosis was analysed by FACS as the percentage of cells positively labelled by Annexin V‐PE. The mean ± SEM is based on replicate experiments (*n* = 3). Statistically significant changes in apoptosis induction (fold increase) are indicated (**p < 0.01; ***p < 0.001). (G) Representative data for apoptosis of Molm14 cells are shown. (H) FC531 potently inhibits M14 cell growth under 1% but not 21% O_2_. Growth inhibition was assessed using MTT cell viability assays. The mean ± SEM is based on replicate experiments (*n* = 3). Statistically significant changes in the percentage of growth inhibition are indicated (*p < 0.05; **p < 0.01). (I) Treatment of M14 cells with FC531 under hypoxic conditions results in a dose dependent increase in CD11b expression. Representative data from flow cytometric analysis (*n* = 2) is shown

In accordance to our findings described above, CA IX and CA XII emerged as promising targets for AML‐directed therapy. To investigate the anti‐leukaemic activity of dual CA IX/XII inhibition, we exposed primary AML samples derived from patients with ND (*n* = 5–6; Figure 4A,B) and R/R (*n* = 3; Figure 4C,D) FLT3 mutated AML to the dual CA IX/XII inhibitors FC531, SLC0111 and CA912 (Figure S1), as well as to Cytarabine and the highly selective FLT3 inhibitor Quizartinib. After 48 h, we performed MTT assays to measure cell proliferation and reduction in cell viability. In accordance to previously published data by Andreucci et al.,^44^ a dose range of 50–200 µM was selected for all three CA inhibitors. As shown in Figure 4A–D, FC531, CA912 and SLC0111 were significantly more effective than Cytarabine and Quizartinib at clinically achievable doses under both 1% and 21% O_2_. The ability of cancer cells to maintain neutral intracellular pH levels under hypoxic stress represents a critical requirement for their survival. Given the central role of CA IX and CA XII in intracellular pH regulation and its druggablity as a trans‐membranous protein, we hypothesised that pharmacologic targeting of CA IX and CA XII will result in strong intracellular acidification and cytotoxicity under low O_2_ conditions. We therefore analysed intracellular pH levels in hypoxia exposed Molm14 cells following treatment with dual CA IX/CA XII inhibition. Because FC531 yielded the greatest effects among the three CA‐targeted agents, we focussed on this compound to further investigate the mechanisms of action of dual CA IX/XII inhibition. Using the pHrodo Red AM fluorescent pH indicator, we found that treatment with FC531 for 48 h led to a significant decrease in intracellular pH levels under hypoxic but not normoxic conditions (Figure 4E, Table 2). Utilising annexin‐V/PI staining we further demonstrated that FC531 substantially induced apoptosis in M14 cells after 48 h under 1% but not 21% O_2_, whereas induction of apoptosis was attenuated by Cytarabine at 1% O_2_ (Figure 4F,G). In line with this observation, MTT assays showed that FC531 exhibited dose dependent, growth inhibitory effects under 1% but not 21% O_2_ in Molm14 cells (IC50 [1%O_2_] = 4.04 μM, 48 h) (Figure 4H), and was more effective than Cytarabine in this respect. Similar results were obtained in Molm13 cells (IC_50_ = 2.85 µM; data not shown). Remarkably, FC531 displayed synergistic toxicity against Molm14 cells when combined with Cytarabine at clinically achievable doses (combination index [CI]: 0.78, 48 h; data not shown). We next determined whether differentiation could be contributing to the overall cytotoxic effects of FC531 against M14 cells. Because FC531 yielded the greatest magnitude of cytotoxicity at 1% O_2_, we focussed on this experimental condition and analysed M14 cells for expression of cell surface markers of differentiation following treatment with FC531. Using decrease of density of CD33 and CD64, and higher expression of CD11b as markers of differentiation, we found that FC531 dose dependently induced the expression of differentiation markers after cells were exposed for 72 h (Figure 4I).

**TABLE 2:** Intracellular pH assessment

Intracellular pH		
O_2_	21%	1%
Untreated	7.40 ± 0.26	7.31 ± 0.51
FC531 1 µM	7.59 ± 0.30	6.80 ± 0.42
FC531 2 µM	7.50 ±0.23	6.32 ± 0.57
FC531 5 µM	7.21 ± 0.42	4.60 ± 1.03

### 3.4 The dual CA IX/ XII inhibitor FC531 is well tolerated and confers anti‐leukaemic activity against FLT3/ITD^+^ AML cells in vivo

**FIGURE 5:**
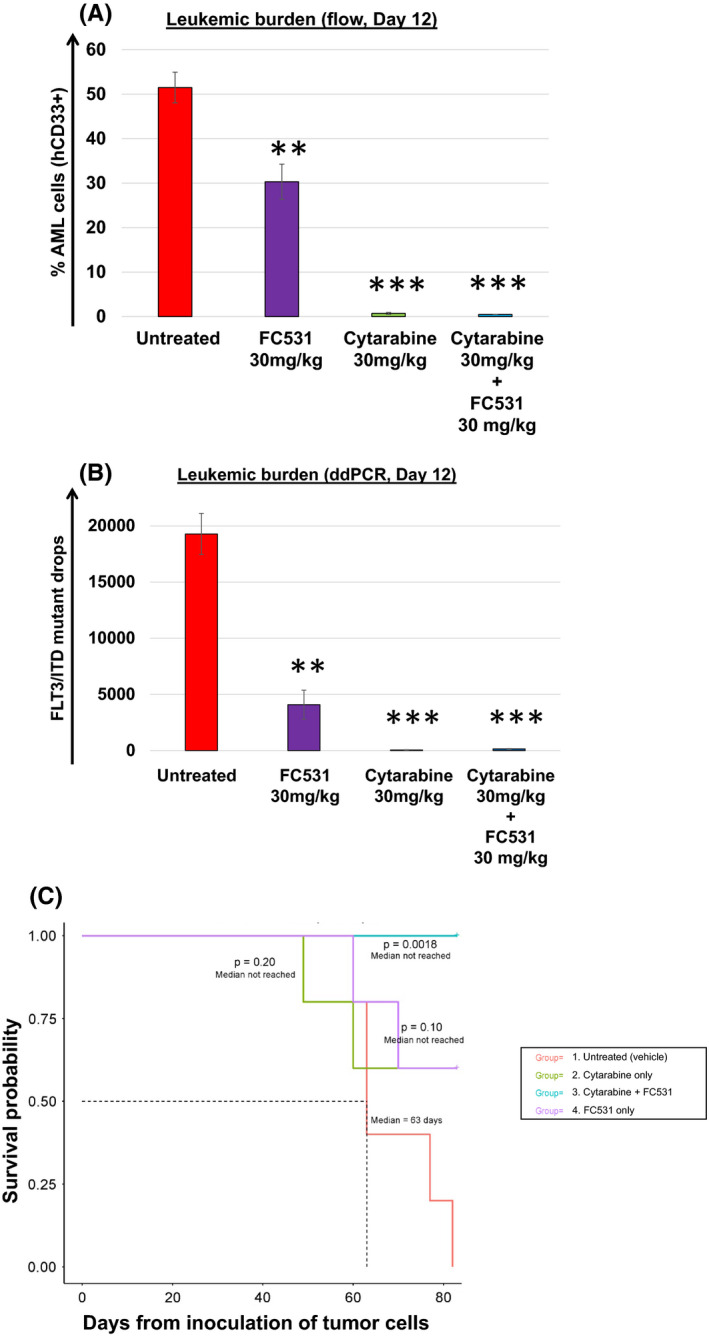
FC531 is well tolerated and confers anti‐leukemic activity against FLT3/ITD^+^ AML cells in PDX models (JAX model J000106134). FC531 demonstrated single agent activity against AML cells in vivo as shown by a significant reduction of leukemic burden after 10 doses. Leukemic burden was assessed via (A) human (h) CD33 positivity per flow cytometry and (B) FLT3/ITD mutant drops as detected per highly sensitive ddPCR. The mean ± SEM is based on replicate experiments (*n* = 5). Statistically significant changes in the change of leukemic burden are indicated (**p < 0.01; ***p < 0.001). (C) Kaplan Meier analysis depicting the survival of mice treated with a 10‐day course (days 1–5 and 8–12) of single agent Cytarabine, single agent FC531 or Cytarabine (days 1–5) followed by FC531 (days 8–12) (as outlined in Table 3). Only combined therapy resulted in statistically improved survival compared to untreated animals (*p* = 0.0018). FC531 only treated mice showed a trend towards improved survival (*p* = 0.1 vs. untreated)

Next, we sought to assess the anti‐leukaemic activity of FC531 in patient derived xenograft (PDX) models.^45^ As shown in Table 3, mice were treated with single agent Cytarabine 30 mg/kg IP (days 1–5 and 8–12), single agent FC531 30 mg/kg IP (days 1–5 and 8–12) or Cytarabine (days 1–5) combined with FC531 (days 8–12, sequential therapy). Mice were monitored for leukaemic burden in peripheral blood (PB) and survival over a course of 82 days. Leukaemic burden was assessed via human (h)CD33 positivity per flow cytometry (Figure 5A) and FLT3/ITD mutant drops as detected per ddPCR (Figure 5B) within 24 h after the last drug dose. Consistent with our in vitro observation, single agent FC531 (Group 4) significantly reduced tumour burden from 51.5 ± 3.4% [untreated, Group 1] to 30.3 ± 3.9% hDC33^+^ (*p* = 0.003; *n* = 5). Combined treatment with Cytarabine (Group 3) led to further reduction in tumour burden (0.5 ± 0.03% hCD33^+^, *p* < 0.01; *n* = 5). Although single agent Cytarabine (Group 2) was similarly effective against AML cells in vivo (0.7 ± 0.2% hCD33^+^, *p* < 0.01; *n* = 5), Cytarabine exposure induced multiple signs of illness, including lethargy, hunched posture and scruffy coat in all animals after 10 days of therapy. In contrast, FC531 was well tolerated in single agent mode (Group 4) and showed only minimal and transient toxicity when combined with a 5‐day course of Cytarabine (Group 3). As demonstrated in Figure 5C, all (5/5) mice in the untreated group had died by day 82 (median survival 63 days). In contrast, combined treatment with FC531 and Cytarabine greatly improved survival with all (5/5) mice surviving for the 82‐day experimental period (*p* < 0.01 vs. untreated, *n* = 5). More than half of the mice (3/5) remained alive in the single agent FC531 and Cytarabine treated groups with a trend towards statistically improved survival when mice were treated with FC531 (*p* = 0.1). Our results demonstrate that FC531 confers anti‐leukaemic activity against AML cells in vivo, is well tolerated in single agent mode and when combined with chemotherapy, and improves survival in PDX models of AML thereby supporting the further investigation of FC531 therapy in clinical trials.

**TABLE 3:** PDX treatment groups

PDX Group	d1‐5	d8‐12
1	PBS (untreated)	PBS (untreated)
2	Cytarabine 30 mg/kg/day i.p.	Cytarabine 30 mg/kg/day i.p.
3	Cytarabine 30 mg/kg/day i.p.	FC531 30 mg/kg/day i.p.
4	FC531 30 mg/kg/day i.p.	FC531 30 mg/kg/day i.p.

### 3.5 CA IX and/or CA XII is expressed in Cytarabine‐residual non‐FLT3 mutated AML cells in vivo

**FIGURE 6:**
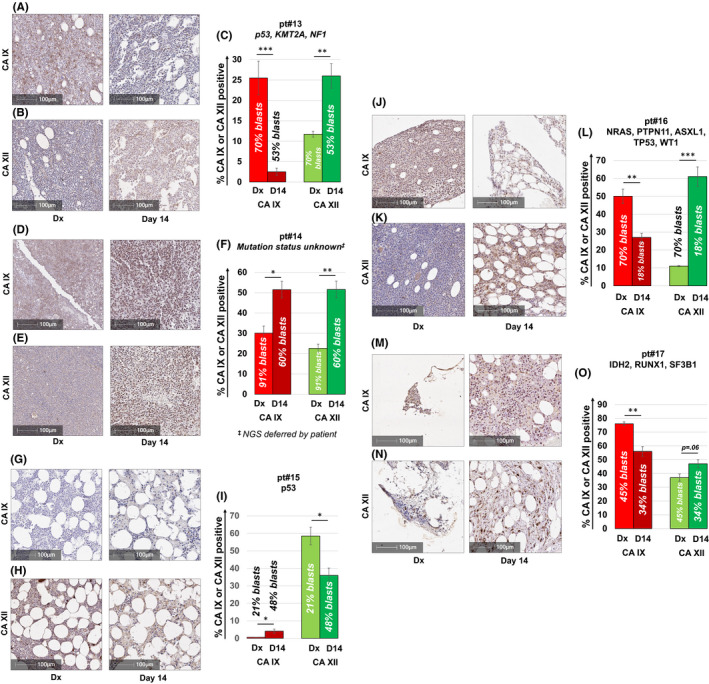
CA IX and/or CA XII is expressed in non‐FLT3 mutated AML patients failing induction chemotherapy. CA IX and XII staining of BM samples from a diverse panel of non‐FLT3 mutated AML patients who had residual disease on their day 14 marrow assessment (A–O). Consistent with data obtained from AML xenograft studies, four out of five patients showed significantly increased CA IX and/or CA XII staining in leukemic blasts remaining after induction chemotherapy. All patients were treated with the “7 + 3” induction regimen. Results shown represent the mean ± SEM % CA IX or XII positivity. Statistically significant changes in the percentage of CA staining are indicated (* <0.05; **p < 0.01; ***p < 0.001). Leukemic blast cell percentages were quantified per clinical flow cytometric immunophenotyping by the Indiana University Health Pathology Laboratory. Corresponding H&E stained sections from the BM are shown in Figure S4A–H

We next assessed the CA IX and XII expression status in non‐FLT3 mutated AML. We therefore stained BM samples from a diverse panel of AML patients (*n* = 5) who had residual disease on their day 14 marrow exam. As shown in Figure 6A–O, four out of five patients showed significantly increased CA IX and/or CA XII staining in leukaemic blasts that escaped chemotherapeutic elimination strongly suggesting that dual CA IX/XII inhibition may be of value as an adjunct to chemotherapy in the management of AML.

## 4 DISCUSSION

Several studies have shown that hypoxia is a critical determinant of drug resistance and disease progression in a wide spectrum of cancers. However, most of the literature in this evolving field of research relies on studies carried out in solid tumours.^46^ The impact of O_2_ deprivation on haematologic malignancies, particularly in AML, is therefore under‐studied. While in solid neoplasms such as breast, pancreatic and renal cell cancers, hypoxia results from a mismatch in supply and consumption of O_2_, a much different mechanism forms the basis of hypoxia in AML. In AML, BM derived neoplastic cells disseminate into the PB where they are exposed to a broad oxygenation window within multiple organs and the vascular system.^7^, ^8^, ^47^ With the intent to achieve a cure, traditional AML‐directed treatments consist of an induction phase, aimed at clearing circulating blasts from the PB and marrow, and a consolidation phase to eliminate residual leukaemia cells and prevent relapse^1^. While effective debulking is frequently achieved, current treatment protocols mostly fail to eliminate residual cells from hypoxic BM niches. Innovative treatment concepts to effectively target drug resistant leukaemia cells that reside in O_2_‐deprived “sanctuaries” therefore represent an unmet need and demand a deeper understanding of the mechanisms involved in this process. It has previously been acknowledged that a major contributor to the slow therapeutic progress in AML^48^ is the difficulty of studying the disease ex vivo and the use of un‐physiologic cell culture techniques.^49^ As AML originates from a population of transformed haematopoietic stem and progenitor cells in the BM, culturing techniques for AML samples have been widely adapted from standardised protocols for haematopoietic stem cell culture, usually carried out in ambient air (normoxia, 21% O_2_). Traditional culture techniques are therefore hyperoxic and not consistent with physiological conditions. In this work, we hypothesised that an in‐depth understanding of hypoxic signalling pathways and the functional consequences of cytotoxic therapy under hypoxic conditions in particular, holds the key for the identification of therapeutic vulnerabilities in AML. In the present study, we explored the molecular responses of FLT3/ITD^+^ AML cells to Cytarabine under O_2_‐controlled conditions in vitro. Taking a transcriptomic and proteomic approach, we found that CA IX and XII are induced in AML cells under hypoxic stress conditions. Intriguingly, CA IX and/or CA XII remained strongly expressed in Cytarabine‐residual cells, as demonstrated per IHC staining in M14 xenograft models and in primary samples obtained from AML patients failing induction chemotherapy. To our knowledge, this is the first report describing the induction of CA IX and XII in AML cells when exposed to low O_2_ levels such as those encountered by leukaemic blasts in clinical settings. Consistent with our observations, some studies have linked the expression of CA IX and XII to the development of drug resistance and poor outcomes.^29^, ^50^, ^51^, ^52^ Specifically, in a study of advanced non‐small cell lung cancer, patients who showed positive CA IX expression levels per IHC after induction chemo‐radiotherapy demonstrated poor survival.^53^ Similarly, Kopecka et al. reported that CA XII expression increased during the acquisition of chemoresistance in colorectal cancer cells and that chemosensitivity could be restored by pharmacological inhibition of CA XII.^19^ As at least CA IX has been reported to be expressed in very low amounts in only a few normal tissues, such as the gastric mucosa,^25^ CA IX inhibition may show relatively few side effects compared to standard anti‐cancer drugs that interact with their target in both healthy and normal tissues. Accordingly, clinical data from early phase CA IX inhibitor trials have shown little toxicity.^54^ We therefore decided to visit the concept of dual CA IX/ XII inhibition in AML. Our screening of three different, dual CA IX/XII inhibitors showed dose dependent activity against a panel of primary FLT3/ITD^+^ AML cells from ND and R/R samples, irrespective of the O_2_ concentration used in the *in vitro* setting. FC531 was more effective than SLC‐0111 and CA912, and exerted its cytotoxic activity via inhibition of cell growth, induction of apoptosis and differentiation. Intriguingly, at the doses tested, the anti‐leukaemic activity of FC531 against Molm14 cells was only observed under hypoxic and not under normoxic conditions. These observations are reminiscent of the studies carried out by Pastorekova and colleagues, who inhibited CA IX/XII enzyme activity with sulphonamides and found that the fluorescent inhibitor only accumulated in hypoxic but not in normoxic tumour cells.^55^, ^56^ The authors explained these observations as consequence of the PG domain of the CA protein, which is open under hypoxic and closes under normoxic conditions. Although higher FC531 doses were used in primary AML cells, the anti‐leukaemic activity of all three dual CA IX/XII inhibitors at 21% O_2_ was surprising. One potential explanation to reconcile this finding is that primary AML cells might retain the hypoxic signature acquired in the O_2_ deprived BM niche, thereby preserving their susceptibility to dual CA IX/ XII inhibition even under normoxic conditions. Similar findings were observed by Msaki et al., who found that disseminated tumour cell tumours with a predilection to the haematopoietic stem cell niche displayed a distinctive hypoxic phenotype that was maintained after 2–4 ex vivo passages under normoxic conditions.^57^ This behaviour indicates that at least in primary cells, the hypoxic signature is maintained by intrinsic features of the leukaemic blasts (e.g. through long term epigenetic activation of hypoxia regulated genes), whereas in established cell lines, such as the Molm14 cells, which have been in culture for decades and are thus well adapted to standard culture conditions, the hypoxic phenotype can be imposed by the O_2_‐deprived environment and is lost when these cells are cultured in ambient air. The significantly decreased tumour burden and improved survival of PDX mice in the single agent and combined treatment arm indicates that FC531 might potentially have a curative effect in AML. Importantly, FC531 treated animals showed minimal to no side effects, which strongly suggests that higher doses and longer treatment courses (e.g. maintenance therapy) might be tolerated in the clinical setting. In support of this concept, a first in human Phase 1 study of SLC‐0111 in patients with advanced solid tumours demonstrated excellent safety and tolerability.^54^ No dose limiting toxicities were reported at doses ≤1000 mg/day administered over a course of 28 days per treatment cycle. In conclusion, our preclinical data indicates that FC531 should be evaluated in clinical trials to assess its activity and toxicity in patients with AML. Despite multiple several new drug approvals in AML since 2017, most agents are available to a subset of patients only.^2^ In addition, hypoxia‐targeted strategies have largely been disappointing to date. For example, a phase 1/2 study of the hypoxia‐activated prodrug PR‐104 in R/R AML only showed modest activity as reported by *Konopleva* et al. In their study, only one patient achieved a complete response. PR‐104 was associated with higher‐grade adverse events and one case of sepsis and death.^29^ While the study by Konopleva et al. clearly provide support for the further evaluation of hypoxia‐targeted strategies, the authors acknowledged that PR‐104 was associated with severe toxicity against haematopoietic stem and progenitor cells. Several clinical trials of PR‐104 have been terminated (e.g. NCT00544674, NCT00862082, NCT00862134), in part due to the low probability of clinically significant results and/or toxicity. The unique mechanism of action of FC531 along with its tolerability suggests that this drug will be a suitable combination partner with traditional and recently approved agents, helping to target residual AML cells hiding in O_2_ deprived niches, thereby decreasing the risk of disease relapse and increasing the chances of a cure.

## CONFLICT OF INTEREST

The authors confirm that there are no conflicts of interest.

## AUTHOR CONTRIBUTIONS

**Fangli Chen:** Conceptualization (lead); formal analysis (lead); methodology (lead); writing–review and editing (lead). **Emilia Licarete:** Formal analysis (supporting); methodology (supporting); writing–review and editing (supporting). **Xue Wu:** Formal analysis (supporting); writing–review and editing (supporting). **Daniela Petrusca:** Formal analysis (supporting); writing–review and editing (supporting). **Callista Maguire:** Methodology (supporting). **Max Jacobsen:** Formal analysis (supporting); methodology (supporting). **Austyn Colter:** Formal analysis (supporting); methodology (supporting). **George E. Sandusky:** Formal analysis (supporting); methodology (supporting); writing–review and editing (supporting). **Magdalena Czader:** Formal analysis (supporting); methodology (supporting). **Maegan L. Capitano:** Formal analysis (supporting); methodology (supporting). **James P. Ropa:** Formal analysis (supporting); methodology (supporting). **H. Scott Boswell:** Formal analysis (supporting); writing–review and editing (supporting). **Fabrizio Carta:** Resources (supporting). **Claudiu T. Supuran:** Resources (supporting). **Brian Parkin:** Formal analysis (supporting); methodology (supporting). **Melissa L. Fishel:** Formal analysis (supporting); methodology (supporting). **Heiko Konig:** Conceptualization (lead); formal analysis (lead); investigation (lead); methodology (lead); supervision (lead); validation (lead); writing–original draft (lead); writing–review and editing (lead).

## Supporting information

Fig S1‐S4

## Data Availability

The data that support the findings of this study are available from the corresponding author upon reasonable request.
