# Supplementary material for: Pharmacological inhibition of Carbonic Anhydrase IX and XII to enhance targeting of acute myeloid leukaemia cells under hypoxic conditions
Source: J Cell Mol Med. 2021 Nov 17;25(24):11039–52. doi: 10.1111/jcmm.17027 (PMC8650039; doi:10.1111/jcmm.17027)
Supplement: Supplementary file 1 — Fig S1‐S4 [file JCMM-25-11039-s001.pdf]

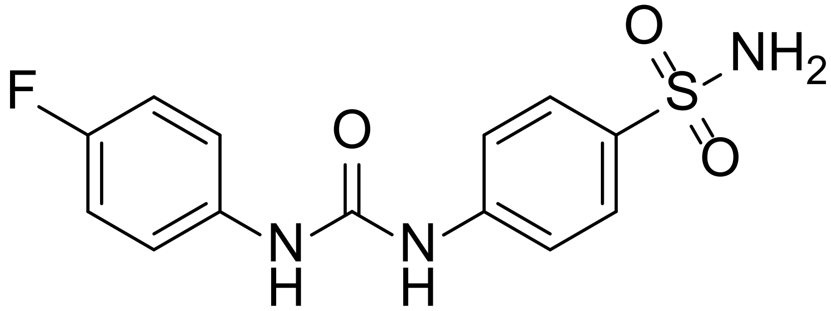

SLC-0111

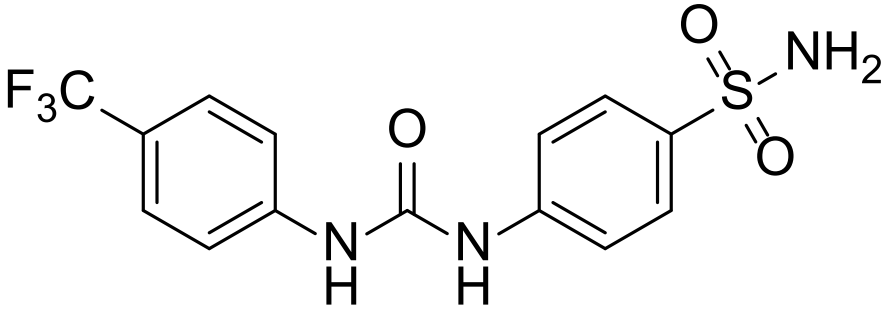

FC531

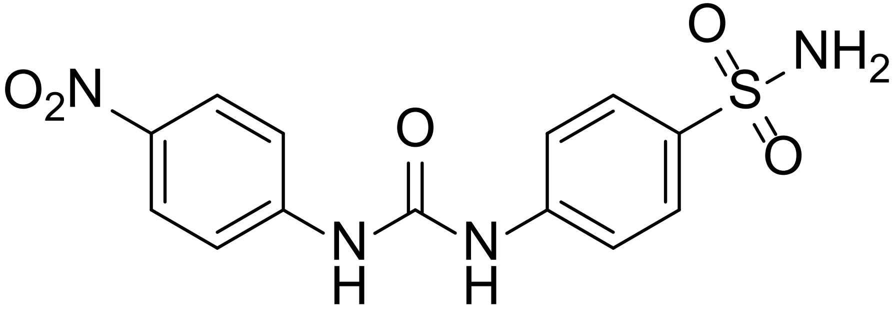

CA912

Supplemental Figure 1: Chemical structures of compounds considered in this study.

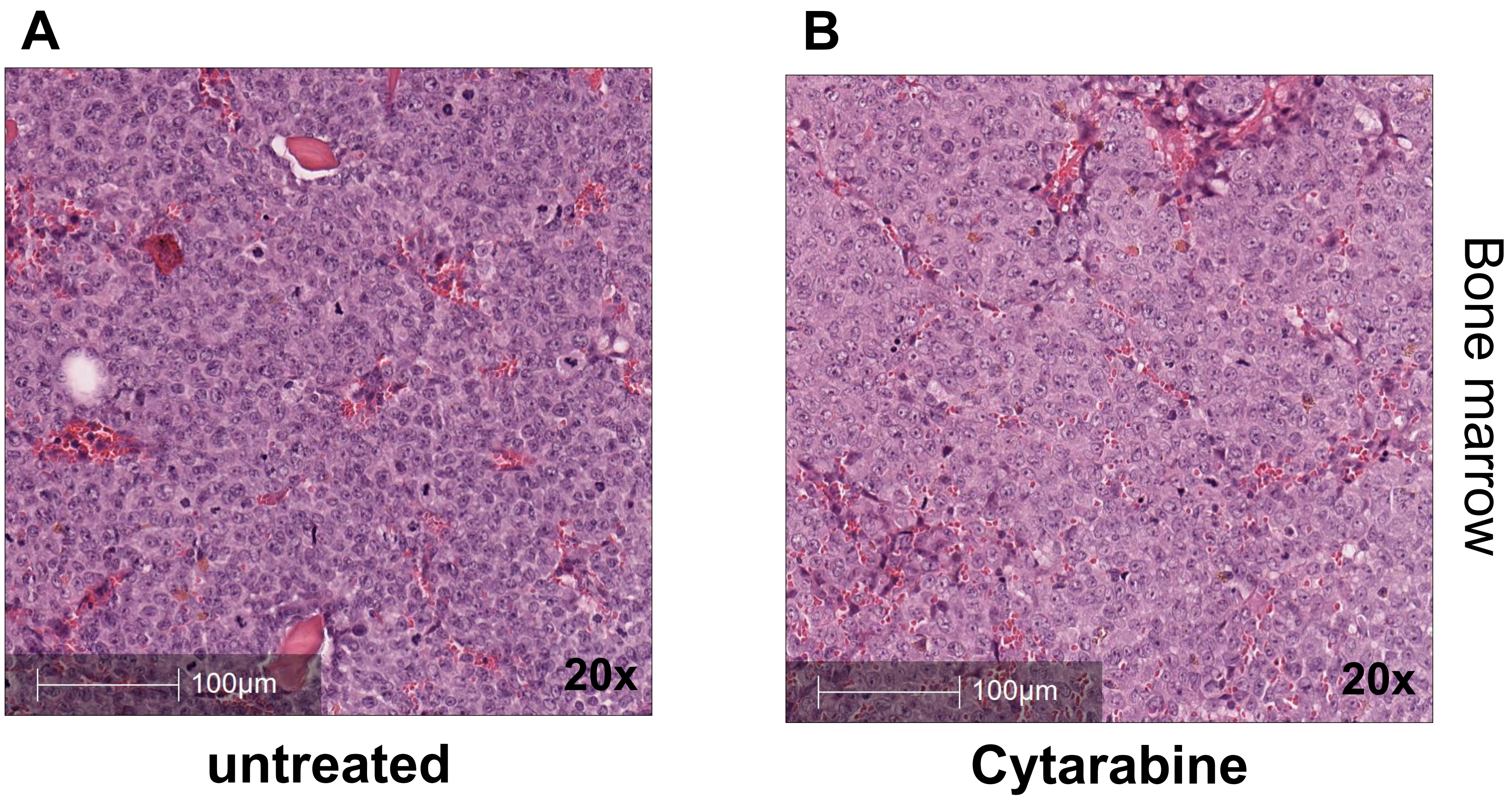

Supplemental Figure 2: Representative H & E stained tissue sections from the BM (A, B) and spleen (C, D) from M14 xenograft studies.

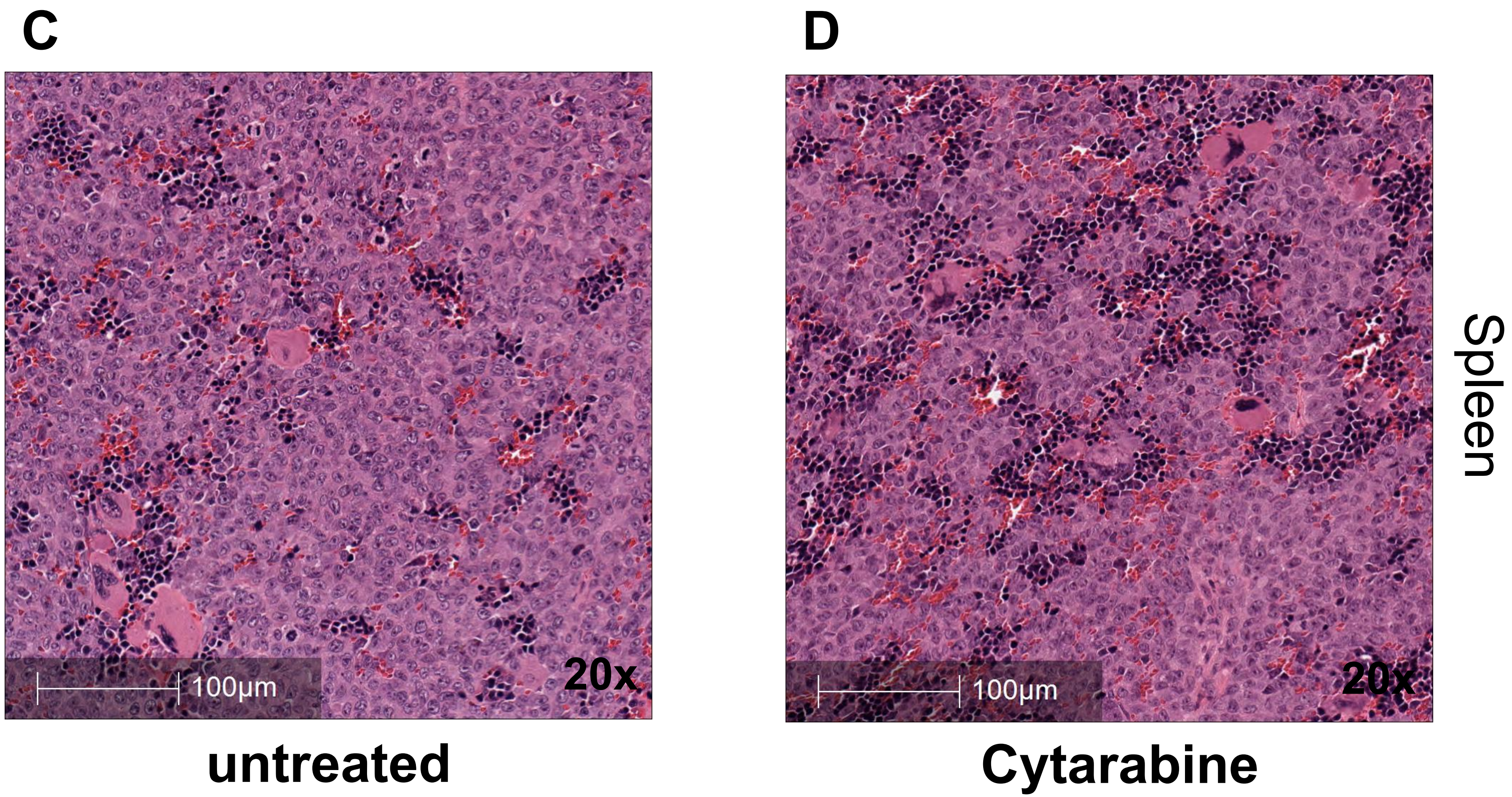

Supplemental Figure 2: Representative H & E stained tissue sections from the BM (A, B) and spleen (C, D) from M14 xenograft studies.

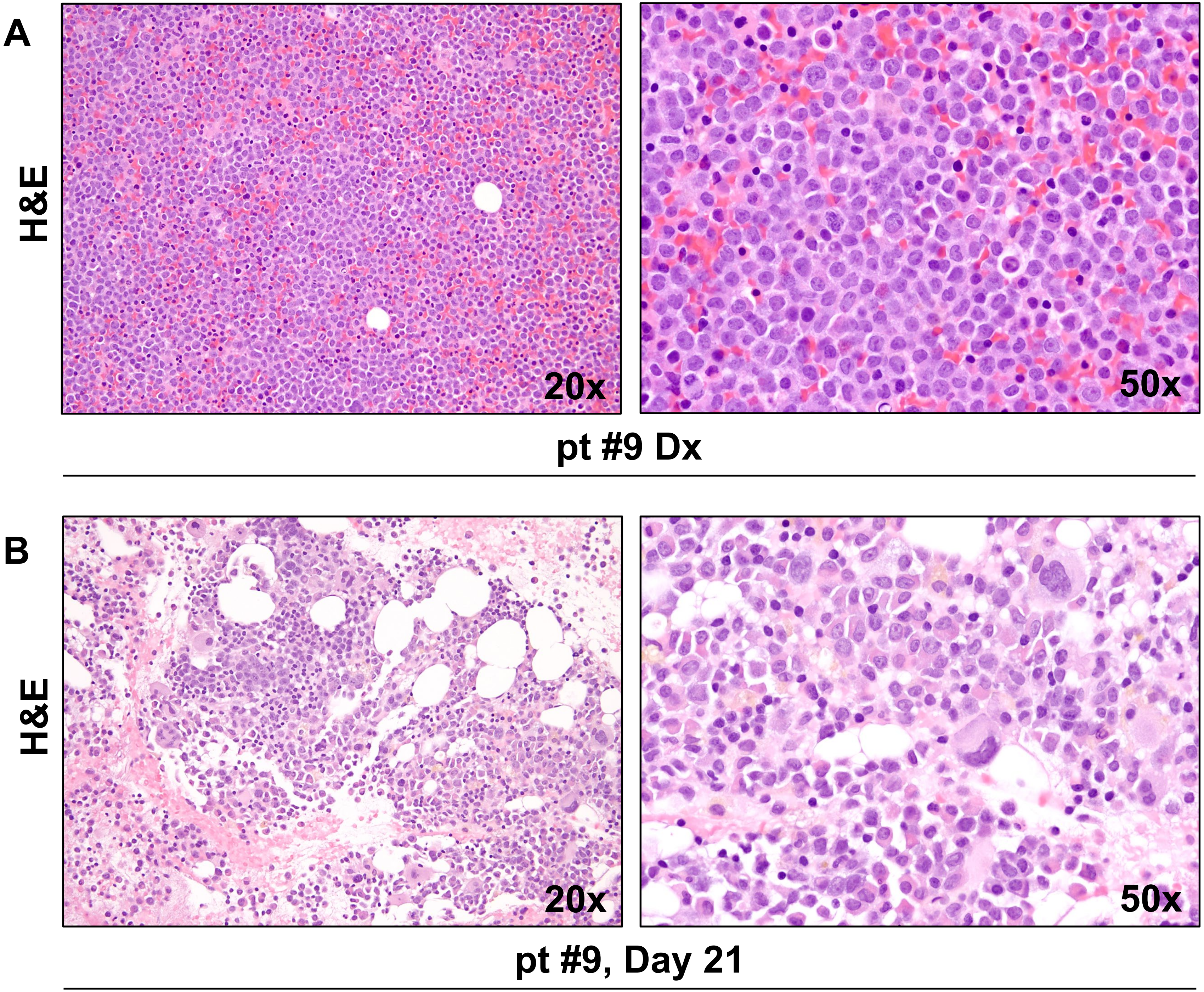

Supplemental Figure 3: Representative H & E stained human BM tissue sections from FLT3-mutated AML patients (see Table 1 for patient characteristics).

C

H&E

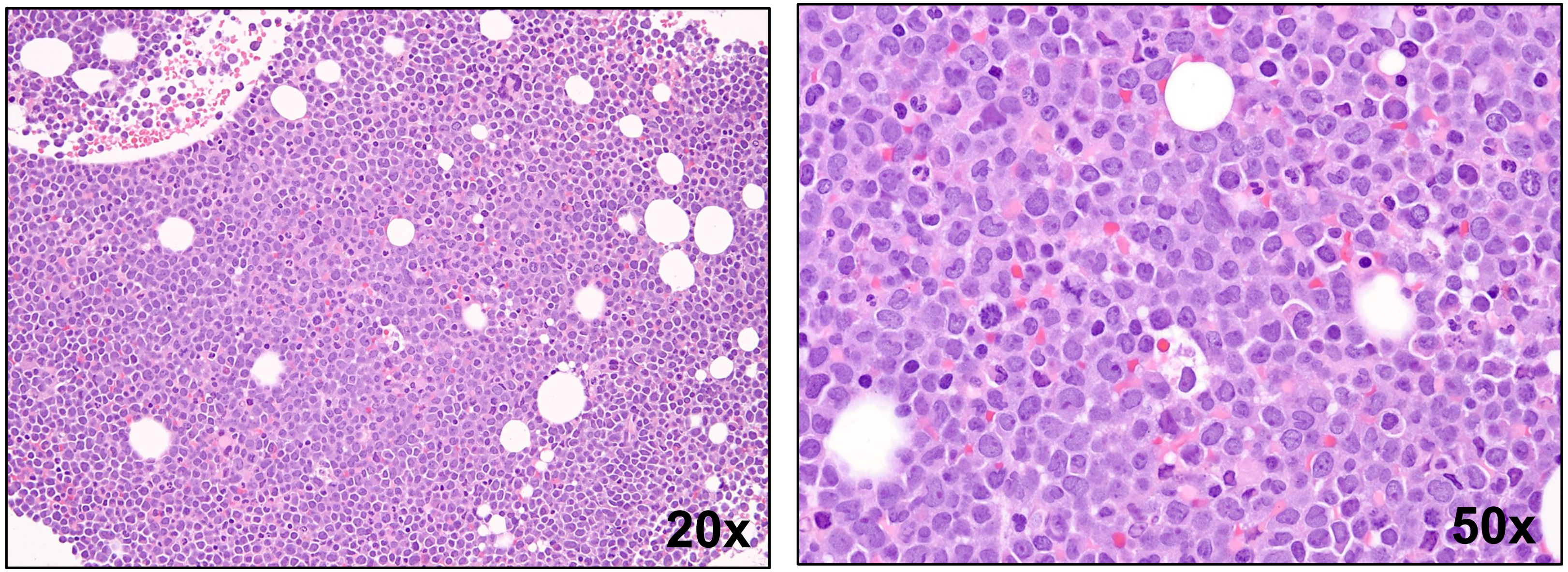

pt #10 Dx

D

H&E

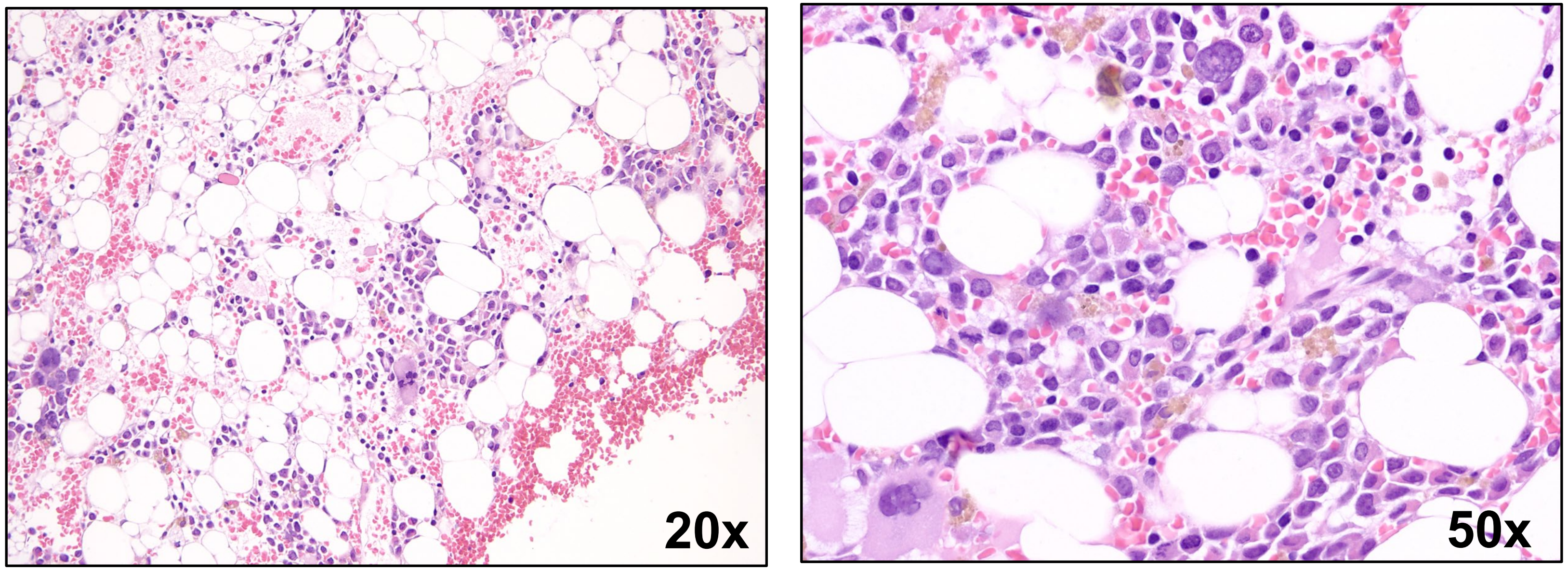

pt #10, Day 21

Supplemental Figure 3: Representative H & E stained human BM tissue sections from FLT3-mutated AML patients (see Table 1 for patient characteristics).

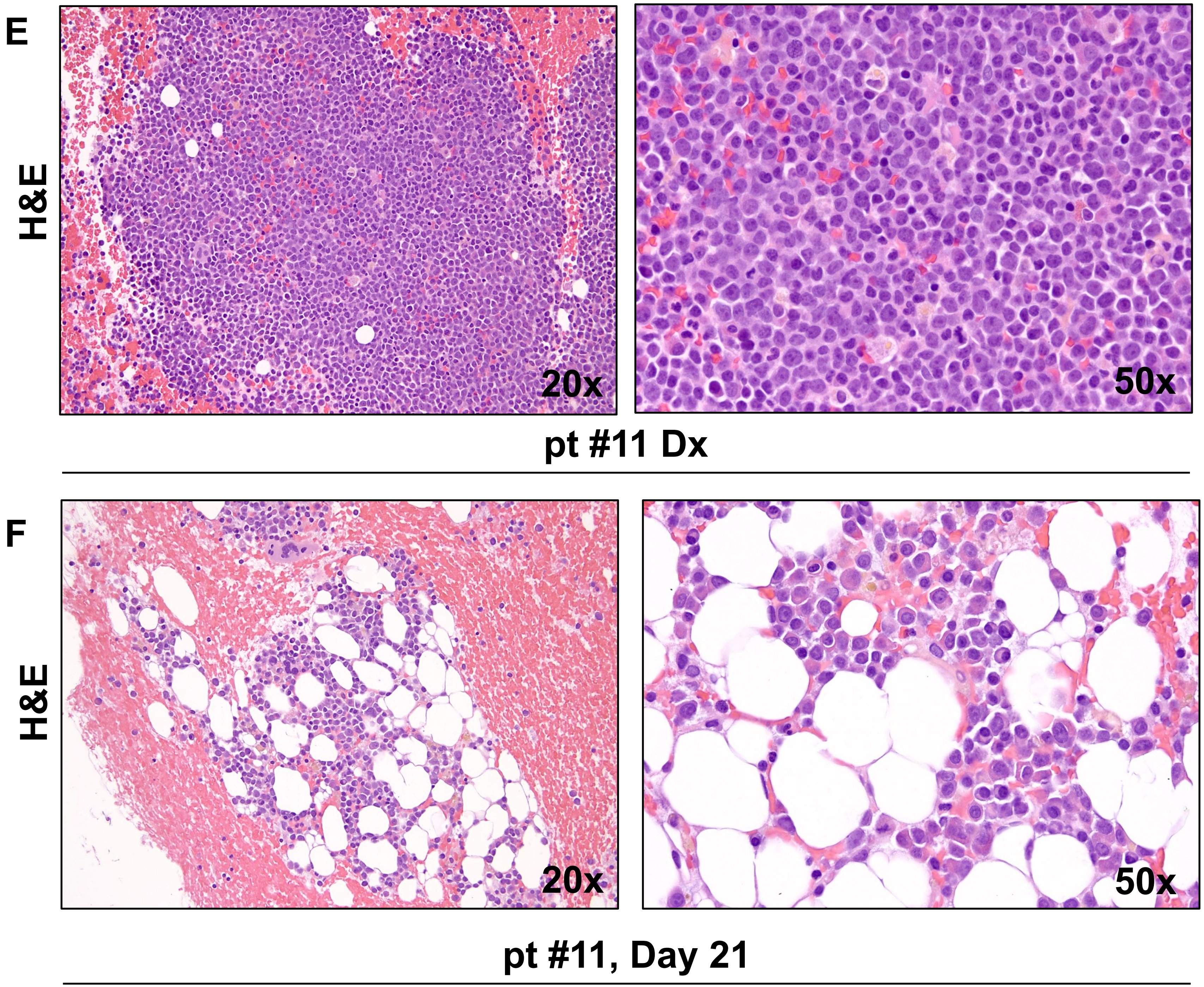

Supplemental Figure 3: Representative H & E stained human BM tissue sections from FLT3-mutated AML patients (see Table 1 for patient characteristics).

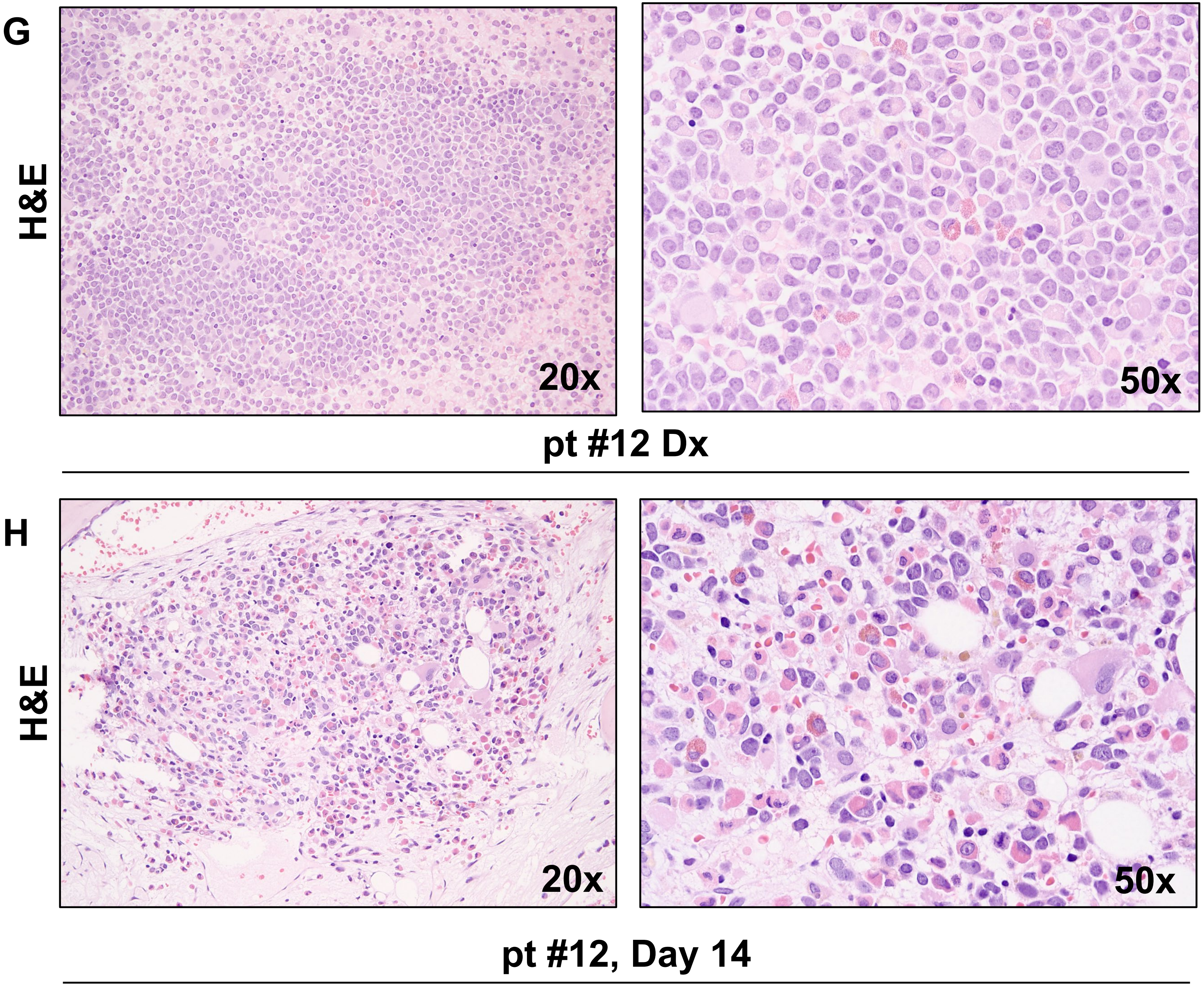

Supplemental Figure 3: Representative H & E stained human BM tissue sections from FLT3-mutated AML patients (see Table 1 for patient characteristics).

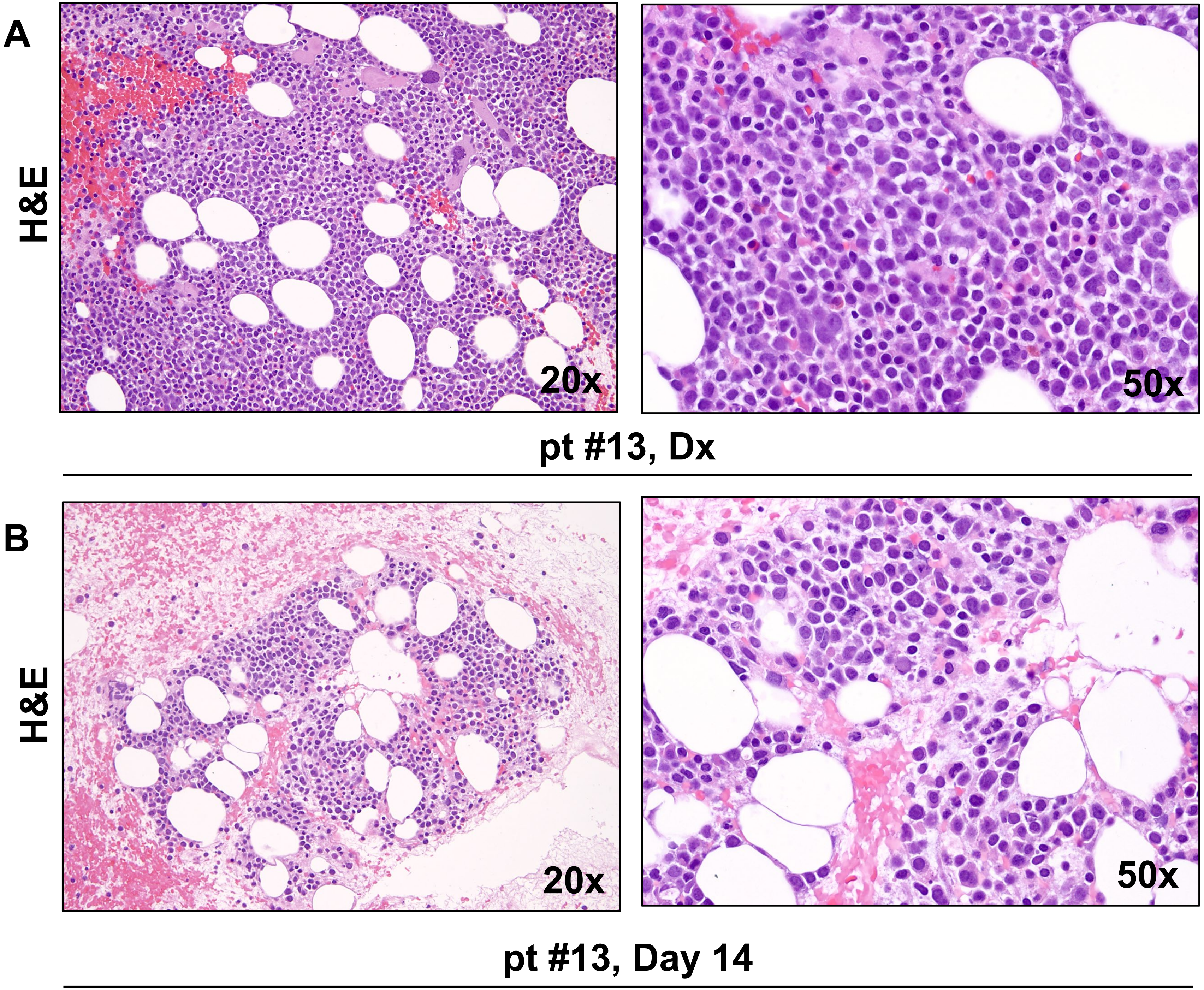

Supplemental Figure 4: Representative H & E stained human BM tissue sections from non-FLT3-mutated AML patients (see Table 1 for patient characteristics). Note: No tissue was available for H&E staining for patient #17.

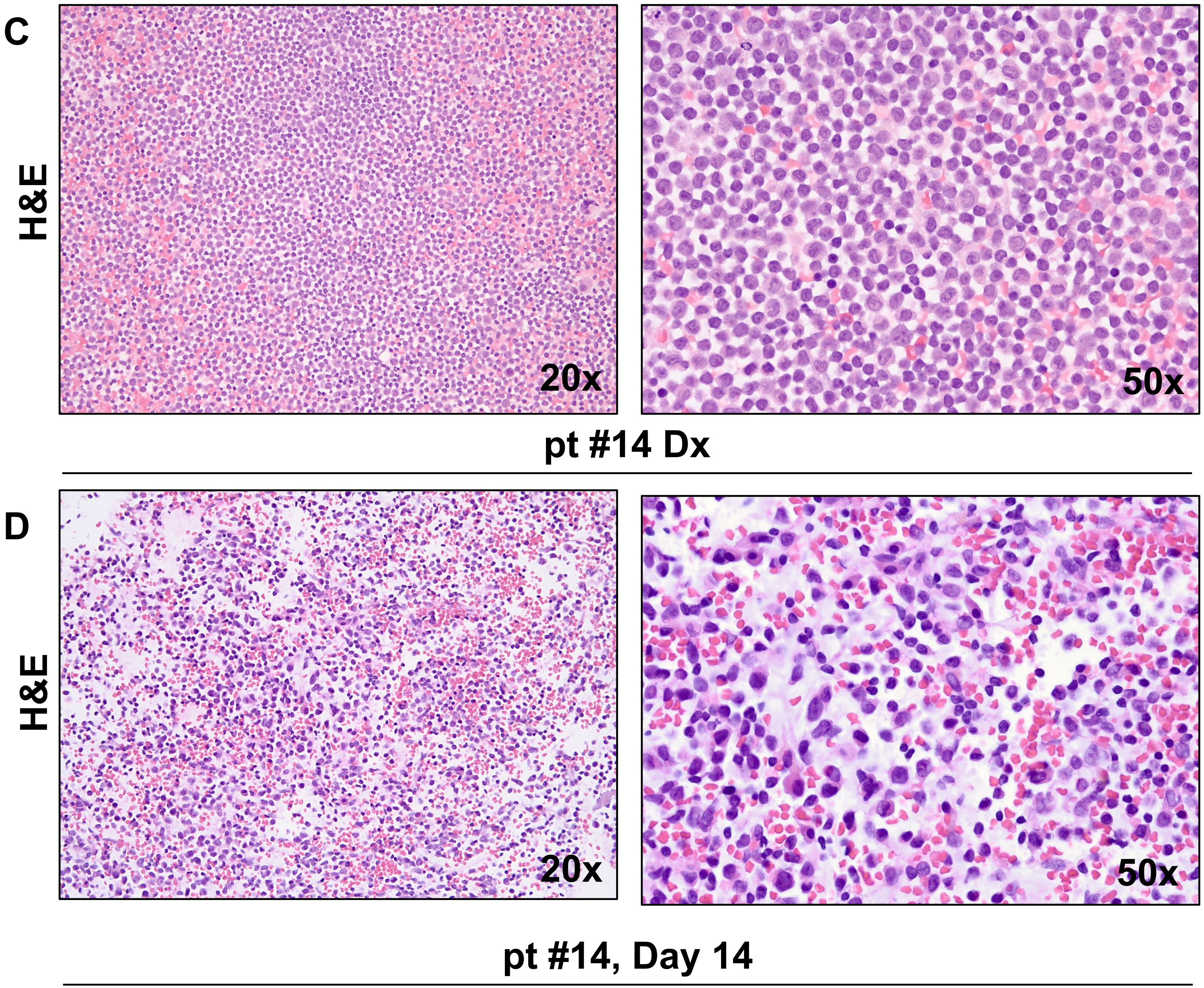

Supplemental Figure 4: Representative H & E stained human BM tissue sections from non-FLT3-mutated AML patients (see Table 1 for patient characteristics). Note: No tissue was available for H&E staining for patient #17.

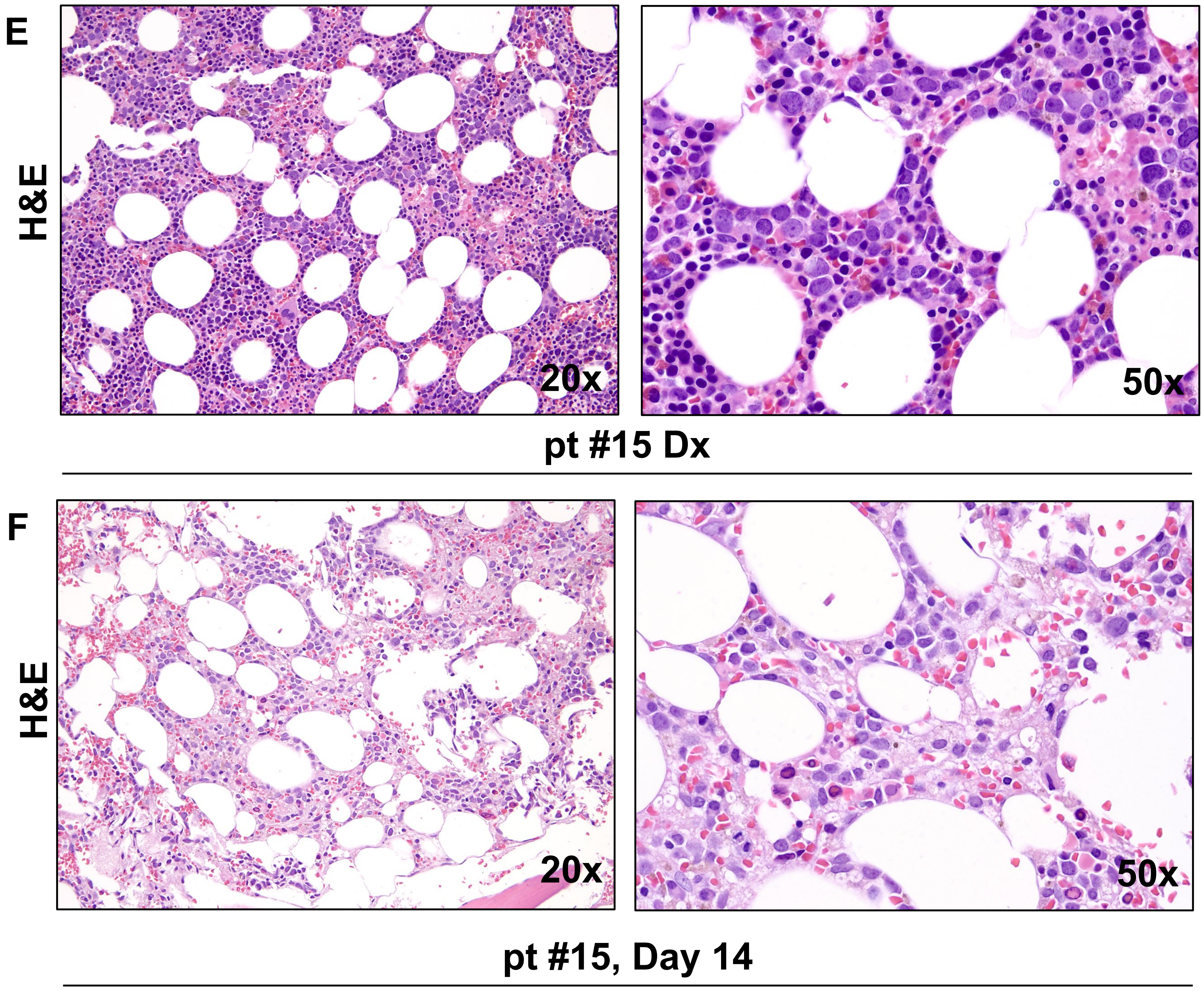

Supplemental Figure 4: Representative H & E stained human BM tissue sections from non-FLT3-mutated AML patients (see Table 1 for patient characteristics). Note: No tissue was available for H&E staining for patient #17.

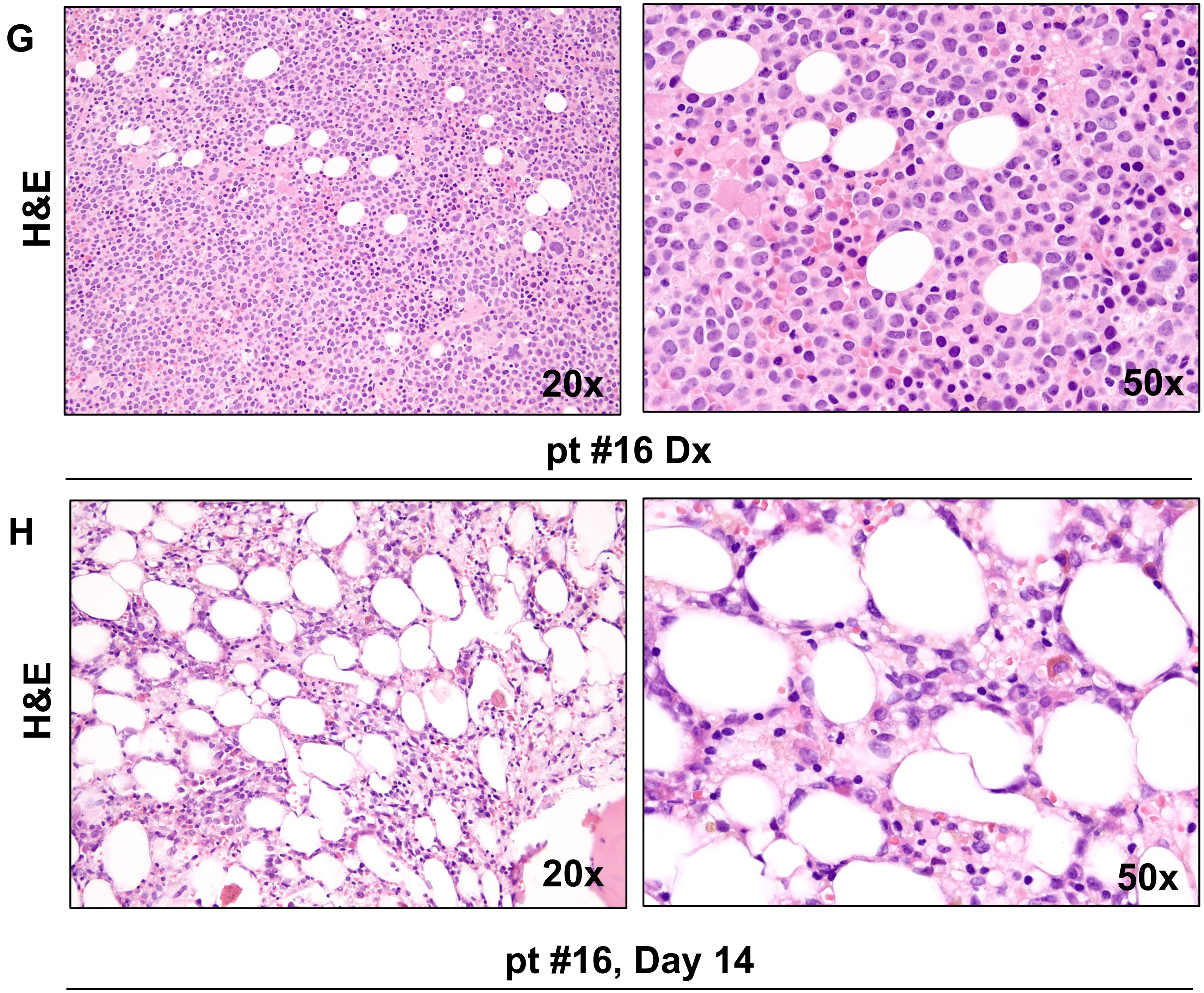

Supplemental Figure 4: Representative H & E stained human BM tissue sections from non-FLT3-mutated AML patients (see Table 1 for patient characteristics). Note: No tissue was available for H&E staining for patient #17.
